# New Insights Into a Classification-Based Microvascular Invasion Prediction Model in Hepatocellular Carcinoma: A Multicenter Study

**DOI:** 10.3389/fonc.2022.796311

**Published:** 2022-03-31

**Authors:** Wei Xu, Yonggang Wang, Zhanwei Yang, Jingdong Li, Ruineng Li, Fei Liu

**Affiliations:** ^1^ Department of Hepatobiliary Surgery, Hunan Provincial People’s Hospital, The First Hospital Affiliated with Hunan Normal University, Changsha, China; ^2^ Department of Hepatobiliary Surgery, Affiliated Hospital of North Sichuan Medical College, Nanchong, China; ^3^ Department of Hepatobiliary Surgery, Xiangtan Central Hospital, Xiangtan, China

**Keywords:** hepatocellular carcinoma (HCC), hepatectomy, microvascular invasion (MVI), predicting model, nomogram

## Abstract

**Background and Aims:**

Most microvascular invasion (MVI)-predicting models have not considered MVI classification, and thus do not reflect true MVI effects on prognosis of patients with hepatocellular carcinoma (HCC). We aimed to develop a novel MVI-predicting model focused on MVI classification, hoping to provide useful information for clinical treatment strategy decision-making.

**Methods:**

A retrospective study was conducted with data from two Chinese medical centers for 800 consecutive patients with HCC (derivation cohort) and 250 matched patients (external validation cohort). MVI-associated variables were identified by ordinal logistic regression. Predictive models were constructed based on multivariate analysis results and validated internally and externally. The models’ discriminative ability and calibration ability were examined.

**Results:**

Four factors associated independently with MVI: tumor diameter, tumor number, serum lactate dehydrogenase (LDH) ≥ 176.58 U/L, and γ-glutamyl transpeptidase (γ-GGT). Area under the curve (AUC)s for our M2, M1, and M0 nomograms were 0.864, 0.648, and 0.782. Internal validation of all three models was confirmed with AUC analyses in D-sets (development datasets) and V-sets (validation datasets) and C-indices for each cohort. GiViTI calibration belt plots and Hosmer-Lemeshow (HL) chi-squared calibration values demonstrated good consistency between observed frequencies and predicted probabilities for the M2 and M0 nomograms. Although the M1 nomogram was well calibrated, its discrimination was poor.

**Conclusion:**

We developed and validated MVI prediction models in patients with HCC that differentiate MVI classification and may provide useful guidance for treatment planning.

## Introduction

Primary liver cancer is the sixth most commonly diagnosed cancer and the third leading cause of cancer death worldwide, with hepatocellular carcinoma (HCC) comprising 75~85% of primary liver cancer cases (1). Chronic hepatitis B virus (HBV) infection has been identified as the predominant determinant of HCC risk in China (1, 2). Hepatectomy and liver transplantation are first-line potentially curative therapies for HCC (3). Although there have been advances in HCC management over the past decade, overall survival has not improved significantly, and long-term prognoses remain poor for most patients (4).

The concept of microvascular invasion (MVI) describes the histologic presence of malignant cells in tumor-adjacent microvessels. MVI is thought to capture local and distant metastasis processes and has been shown to be a significant risk factor for both early recurrence and poor prognosis after tumor resection or liver transplantation (5). Thus, MVI diagnosis could, theoretically, affect therapeutic planning, including decisions regarding the width of margins to be taken during hepatectomy, microwave/radiofrequency ablation, and postoperative adjunct treatment modalities. However, currently, MVI can only be confirmed definitively by postoperative pathology, limiting the role of preoperative evaluation in surgical planning and patient prognosis (6, 7).

Although the notion that MVI affects prognosis adversely has gained acceptance over time, there is not a consensus regarding the precise relationship between MVI and patient outcomes (8, 9). Our previous findings indicated that a simple distinction of MVI presence or absence, as has been most commonly done in MVI-related prediction modeling studies (6, 7), is inadequate for predicting early recurrence after curative hepatectomy for HCC (10). Some recent prediction models in the literature have classified MVI in some detail (11–17), but there is not yet a universally recognized consensus MVI classification scheme.

The purpose of this study was to identify clinical variables that are significantly associated with particular classifications of MVI. We employed data from independent clinical cohorts of patients receiving hepatectomies in one of two Chinese Medical Centers to develop prediction models for predicting MVI classes. The models were validated externally.

## Materials and Methods

### Study Design and Participants

A retrospective study was conducted with data from two Chinese Medical Centers: Hunan Provincial People’s Hospital (HPPH) and Affiliated Hospital of North Sichuan Medical College (NSMC). The institutional review boards of both medical centers approved the study (HPPH approval no: 2020-018; NSMC approval no: 2020-055). Given the purely observational nature of the study and no patients being contacted for the purpose of this study, the need for written informed consent was waived by the Institutional Review Board. Patient privacy was ensured, and the data were anonymized or maintained with confidentiality. All procedures were performed in accordance with the 1964 Helsinki Declaration and its later amendments or comparable ethical standards. The study was conducted in accordance with the Transparent Reporting of a Multivariable Prediction Model for Individual Prognosis or Diagnosis statement for reporting multivariable prediction model derivation and validation (18). Neither patients nor the public were involved in the design, conduct, reporting, or dissemination plans of this research.

A derivation cohort was formed from 800 eligible consecutive patients treated at Hunan Provincial People’s Hospital with the inclusion criterion of a histological diagnosis of HCC following an initial hepatectomy performed between January 2015 and September 2019. An external validation cohort was formed from 250 consecutive matched patients operated on at the Affiliated Hospital of North Sichuan Medical College between January 2018 and September 2019 with otherwise the same inclusion criterion. The exclusionary criteria were: (a) a pathology finding other than HCC; (b) hepatectomy of recurrent HCC; (c) a prior hepatectomy for HCC at another hospital;(d) previous history of other type of cancer; and (e) other treatment (such as transcatheter arterial chemoembolization, radiotherapy) before hepatectomy. The clinical characteristics of the patients are summarized in **Table 1**.

**Table 1:** Demographic and clinicopathological characteristics of the derivation and external validation cohorts.

Characteristic (N)	Derivation cohort (N = 800) N (%) or median (range)		External validation cohort (N = 250) N (%) or median (range)	*P*
**Gender**				0.528 *^a^*
Female (134)	105 (13.1)	29 (11.6)		
Male (916)	695 (86.9)	221 (88.4)		
**Age, years**				0.768 *^b^*
≤40 (140)	106 (13.3)	34 (13.6)		
40–60 (557)	428 (53.5)	129 (51.6)		
≥60 (353)	266 (33.3)	87 (34.8)		
**Hepatitis virus type**				0.003 *^b^*
None (111)	94 (11.8)	17 (6.8)		
Hepatitis B (901)	683 (85.4)	218 (87.2)		
Hepatitis C (34)	20 (2.5)	14 (5.6)		
Hepatitis B+C (4)	3 (0.4)	1 (0.4)		
**Antiviral therapy**				0.192 *^a^*
No (935)	718 (89.8)	217 (86.8)		
Yes (115)	82 (10.3)	33 (13.2)		
**HBsAg (+)**				0.674 *^a^*
No (190)	147 (18.4)	43 (17.2)		
Yes (860)	653 (81.6)	207 (82.8)		
**Spontaneous HBsAg seroclearance**				0.656 *^a^*
Yes (126)	94 (11.8)	32 (12.8)		
No (924)	706 (88.3)	218 (87.2)		
**HBV-DNA < 100 IU/ml**				0.135 *^a^*
No (618)	481 (60.1)	137 (54.8)		
Yes (432)	319 (39.9)	113 (45.2)		
**Child-Pugh grade**				0.075 *^a^*
A (1,009)	764 (95.5)	245 (98.0)		
B (41)	36 (4.5)	5 (2.0)		
**Tumor MD, cm**				0.032 *^b^*
≤2 (245)	57 (7.1)	11 (4.4)		
2-5 (171)	304 (38.0)	84 (33.6)		
≥5 (78)	439 (54.9)	155 (62.0)		
**Tumor number**				0.734 *^b^*
1 (734)	558 (69.8)	176 (70.4)		
2 (128)	95 (11.9)	33 (13.2)		
3 (31)	24 (3.0)	7 (2.8)		
≥4 (157)	123 (15.4)	34 (13.6)		
**Tumor location**				0.171 *^b^*
Left (241)	187 (23.4)	54 (21.6)		
Right (711)	546 (68.3)	165 (66.0)		
Bilobar (98)	67 (8.4)	31 (12.4)		
**With SR**				0.991 *^a^*
No (941)	717 (89.6)	224 (89.6)		
Yes (109)	83 (10.4)	26 (10.4)		
**PVTT**				0.178 *^b^*
None (941)	723 (90.4)	218 (87.2)		
Vp1 (7)	3 (0.4)	4 (1.6)		
Vp2 (32)	22 (2.8)	10 (4.0)		
Vp3 (56)	41 (5.1)	15 (6.0)		
Vp4 (14)	11 (1.4)	3 (1.2)		
**BDTT**				0.584 *^b^*
None (1006)	765 (95.6)	241 (96.4)		
Microscopic (15)	11 (1.4)	4 (1.6)		
Macroscopic (29)	24 (3.0)	5 (2.0)		
**HVTT**				0.055 *^b^*
None (1012)	776 (97.0)	236 (94.4)		
Vv1 (3)	3 (0.4)	0 (0)		
Vv2 (30)	17 (2.1)	13 (5.2)		
Vv3 (5)	4 (0.5)	1 (0.4)		
**IVCTT**				>0.999 *^c^*
No (1043)	794 (99.3)	249 (99.6)		
Yes (7)	6 (0.8)	1 (0.4)		
**Tumor capsular**				0.447 *^b^*
No (639)	492 (61.5)	147 (58.8)		
Incomplete (16)	12 (1.5)	4 (1.6)		
Complete (395)	296 (37.0)	99 (39.6)		
**Lymph node metastasis**				0.709 *^c^*
No (1040)	793 (99.1)	247 (98.8)		
Yes (10)	7 (0.9)	3 (1.2)		
**AJCC-TNM stage**				0.982 *^b^*
IA (58)	48 (6.0)	10 (4.0)		
IB (416)	319 (39.9)	97 (38.8)		
II (245)	190 (23.8)	55 (22.0)		
IIIA (132)	102 (12.8)	30 (12.0)		
IIIB (180)	128 (16.0)	52 (20.8)		
IVA (9)	6 (0.8)	3 (1.2)		
IVB (10)	7 (0.9)	3 (1.2)		
**BCLC stage**				0.076 *^b^*
0 (40)	34 (4.3)	6 (2.4)		
A (618)	474 (59.3)	144 (57.6)		
B (201)	160 (20.0)	41 (16.4)		
C (189)	131 (16.4)	58 (23.2)		
D (2)	1 (0.1)	1 (0.4)		
**Alpha fetoprotein (μg/L)**	141.8 (0.2–981900.0)	133.8 (0.3–463500.0)		0.317 *^d^*
**Metavir inflammation grade**				0.760 *^b^*
A0 (75)	65 (8.1)	10 (4.0)		
A1 (701)	523 (65.4)	178 (71.2)		
A2 (252)	192 (24.0)	60 (24.0)		
A3 (22)	20 (2.5)	2 (0.8)		
**Metavir fibrosis grade**				0.029 *^b^*
F0 (18)	18 (2.3)	0 (0)		
F1 (65)	54 (6.8)	11 (4.4)		
F2 (414)	315 (39.4)	99 (39.6)		
F3 (267)	208 (26.0)	59 (23.6)		
F4 (286)	205 (25.6)	81 (32.4)		
**Edmondson-Steiner stage**				0.750 *^b^*
I (26)	24 (3.0)	2 (0.8)		
II (333)	255 (31.9)	78 (31.2)		
III (598)	444 (55.5)	154 (61.6)		
IV (93)	77 (9.6)	16 (6.4)		
**MVI**				<0.001 *^b^*
M0 (463)	382 (47.8)	81 (32.4)		
M1 (348)	257 (32.1)	91 (36.4)		
M2 (239)	161 (20.1)	78 (31.2)		

a Pearson x^2^.

b Wilcoxon rank sum.

c Fisher’s exact.

d Mann-Whitney U.

SR, spontaneous rupture; PVTT, portal vein tumor thrombosis; BDTT, bile duct tumor thrombosis; HVTT, hepatic vein tumor thrombosis; IVCTT, inferior vena cava tumor thrombosis; MVI, microvascular invasion.

### Preoperative Assessment

Patients’ demographic and clinicopathological data were collected according to routine post-admission practices. The latest preoperative laboratory and imaging examination results were used in the present analyses. Portal hypertension was diagnosed according to published criteria (19) and classified as mild, moderate, or severe, as described in detail previously (10). Portal vein tumor thrombosis (PVTT) and hepatic vein tumor thrombosis (HVTT) were graded based on published clinical criteria (20). Bile duct tumor thrombus (BDTT) was classified as previously reported (21).

### Tumor Staging and Pathology

Resected tumors were staged postoperatively in accordance with the American Joint Committee on Cancer’s TNM staging system (8^th^ edition) (22) and the Barcelona Clinic Liver Cancer system (23). Tumor size was defined as the maximum diameter of the pathology specimen. Tumor number was classified as 1, 2, 3, or ≥4. Satellite nodules were defined as microscopic HCC nodules separated from the tumor by at least 2 cm of uninvolved liver parenchyma and included in tumor counts. Tumor cell differentiation was classified according to the Edmondson-Steiner system (24). HCC pathology samples were taken in accordance with a 7-point baseline sample collection protocol (25). MVI was defined as the presence of a cancer cell nest in vessels lined by endothelial cells on microscopy and classified based on the 2015 update of the Evidence-Based Practice Guidelines for Standardized Pathological Diagnosis of Primary Liver Cancer in China (25) as follows: M0 (MVI absent); M1 (≤5 MVI sites, all located within adjacent peritumoral liver tissues ≤ 1 cm from the tumor margin); or M2 (>MVI sites, or the presence of any MVI site(s) within adjacent peritumoral liver tissues > 1 cm away from the tumor margin). MVIs in all tissue specimens were counted by examination of serial sections under a light microscopic. Liver tissue inflammation and fibrosis in non-tumor areas were graded according to the METAVIR scoring system (26). Two senior pathologists with more than 10 years of hepatic pathology experience performed pathological examinations of the surgical specimens. A consensus was reached by discussion for discordant cases.

### Surgical Therapies

Surgery was performed by senior hepatic specialists with ≥ 15 years of clinical experience. HCCs were removed by open or laparoscopic hepatectomy, according to surgical methods as we reported previously (10). PVTT and/or HVTT were removed by tumor thrombectomy or by *en-bloc*-resection of the thrombotic vein(s), with or without post-resection portal/hepatic vein reconstruction. BDTT was addressed with tumor thrombectomy or by *en-bloc*-resection inclusive of the bile duct. An inferior vena cava tumor thrombosis was resected concomitantly with the primary tumor resection after establishing well-controlled total hepatic vascular exclusion.

### Statistical Analysis

Categorical variables, which are reported as numbers of cases with percentages, were analyzed with chi-square or Fisher’s exact tests. Continuous variables, which are reported as medians with minimum-maximum value ranges, were analyzed with Mann-Whitney tests. To assess the predictive values of MVI data, univariate ordinal logistic regression analyses were conducted with derivation cohort data. Continuous variables that were determined to be significant in the univariate analyses were included in follow-up statistical analyses. Otherwise, cut-off values were determined based on the maximum Youden index from receiver operating characteristic (ROC) analysis and then dichotomized into univariate analysis. Variables with *p* values < 0.05 in the univariate analysis were submitted to multivariate analysis in accordance with a stepwise backward-elimination procedure (threshold *p* < 0.05). An MVI prediction nomogram was developed based on the multivariate ordinal logistic regression analysis results.

The predictive performance of the presently constructed MVI prediction models was validated internally with derivation cohort data by 2000 bootstrap re-samplings of 75% of the original data and then validated externally. Discrimination ability was assessed by constructing an area under the ROC curve (AUROC) and estimating an associated C-statistic. AUROC values are reported with 95% confidence intervals (CIs). C-statistic values were calculated as described previously (27). Agreement between the MVI classification model predictions and empirically observed frequencies were assessed with a GiViTI calibration belt plot and the Hosmer-Lemeshow (HL) chi square test of goodness of fit. All statistical analyses were performed in SPSS software (version 23.0; IBM Corporation, Armonk, NY) and R software (version 3.3.0) with the rms package (version 5.1-1; http://www.R-project.org). In all cases, *p* < 0.05 was considered significant.

## Results

### Patient and Clinicopathologic Characteristics

**Figure 1:**
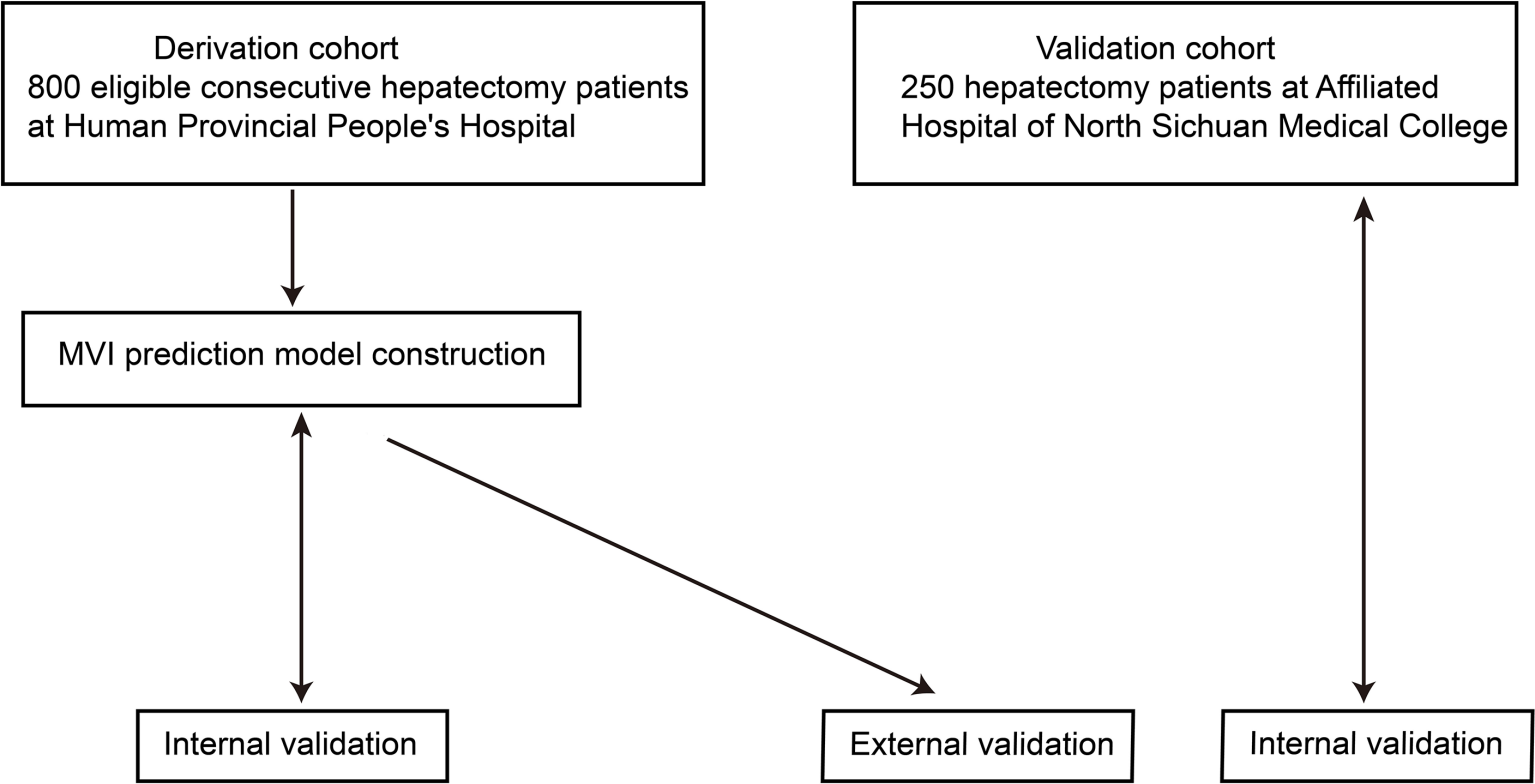
Flow chart overview of the study. The derivation and validation cohorts were gathered during the periods of January 2015–September 2019 and January 2018–September 2019, respectively. Internal validation was conducted with the bootstrap method (2000 samples drawn from 75% of the original data).

A study overview flow diagram is provided in **Figure 1**. The baseline characteristics of all included patients, including the derivation (N = 800) and external validation (N = 250) cohorts, are presented in **Table 1**. All other clinical characteristics were statistically similar between the two groups, with the exceptions of hepatitis virus infection type, tumor maximum diameter and METAVIR grade of background liver fibrosis.

### Predictor Variables for MVI

The cut-off values of continuous variables determined by ROC curves are shown in **Table 2**. The results of univariate ordinal logistic regression analysis of clinical features in the derivation cohort are shown in **Table 3**. In the multivariate analysis of putative predictive factors found to be significant in the univariate analyses, risk factors that independently associated with the presence of MVI were tumor size, number of tumors, serum LDH ≥ 176.58 U/L, and serum γ-GGT (U/L) (**Table 4**).

**Table 2:** Determination of cutoff points for clinical factors determined by ROC curve analysis.

Factor	M1						M2					
	AUC	95%CI	Sig.	CV	Sensitivity (%)	Specificity (%)	AUC	95%CI	Sig.	CV	Sensitivity (%)	Specificity (%)
Tumor MD (cm)	0.564	(0.527,0.600)	0.001	5.55	59.5	50.9	0.687	(0.651,0.724)	0.000	6.95	64.9	64.3
Tumor numbers	0.492	(0.455,0.528)	0.656	—	—	—	0.662	(0.620, 0.704)	0.000	1.5	52.3	76.4
ALT (U/L)	0.446	(0.384,0.508)	0.079	—	—	—	0.543	(0.497, 0.588)	0.066	—	—	—
AST (U/L)	0.488	(0.447,0.529)	0.572	—	—	—	0.482	(0.422,0.543)	0.566	—	—	—
LDH (U/L)	0.491	(0.429,0.553)	0.771	—	—	—	0.670	(0.613, 0.728)	0.000	176.58	86.9	35.2
5’-NT (U/L)	0.468	(0.406,0.529)	0.294	—	—	—	0.544	(0.494,0.593)	0.086	—	—	—
γ-GGT (U/L)	0.476	(0.415,0.536)	0.435	—	—	—	0.456	(0.396,0.516)	0.149	—	—	—
ALB (g/L)	0.553	(0.494,0.612)	0.086	—	—	—	0.514	(0.466, 0.561)	0.576	—	—	—
GLB (g/L)	0.467	(0.406,0.529)	0.286	—	—	—	0.462	(0.402, 0.522)	0.212	—	—	—
TBIL (mmol/L)	0.452	(0.391,0.513)	0.120	—	—	—	0.561	(0.495, 0.628)	0.077	—	—	—
DBIL (mmol/L)	0.485	(0.425,0.545)	0.622	—	—	—	0.560	(0.493, 0.627)	0.086	—	—	—
RBC (×10^12^)	0.540	(0.480,0.600)	0.192	—	—	—	0.557	(0.486, 0.627)	0.102	—	—	—
WBC (×10^9^)	0.504	(0.444,0.564)	0.900	—	—	—	0.534	(0.467, 0.602)	0.321	—	—	—
L	0.519	(0.458,0.580)	0.538	—	—	—	0.475	(0.411, 0.539)	0.469	—	—	—
NLR	0.485	(0.423,0.548)	0.637	—	—	—	0.565	(0.501, 0.628)	0.063	—	—	—
Hb (g/L)	0.506	(0.447,0.565)	0.851	—	—	—	0.517	(0.446, 0.588)	0.628	—	—	—
PLT (×10^12^)	0.528	(0.469,0.588)	0.359	—	—	—	0.596	(0.550, 0.643)	0.000	162.5	61.0	55.3
PA (mg/L)	0.531	(0.471,0.592)	0.309	—	—	—	0.431	(0.383, 0.480)	0.006	—	—	—
ALP (U/L)	0.464	(0.402,0.525)	0.238	—	—	—	0.565	(0.497, 0.633)	0.061	—	—	—
AFP (μg/L)	0.526	(0.486,0.565)	0.207	—	—	—	0.632	(0.589, 0.676)	0.000	11.08	85.7	32.4
HBsAg quantity	0.535	(0.475,0.594)	0.263	—	—	—	0.502	(0.434, 0.570)	0.947	—	—	—
HBV-DNA (IU/ml)	0.518	(0.478,0.557)	0.389	—	—	—	0.515	(0.443, 0.586)	0.676	—	—	—

AUC: Area under the curve; CV: Cutoff point value; ALT, glutamic pyruvic transaminase; AST, aspartate aminotransferase; LDH, lactate dehydrogenase; NT, nucleotidase; γ-GGT, γ-glutamyl transpeptidase; ALB, albumin; GLB, globulin; TBIL, total bilirubin; DBIL, direct bilirubin; RBC, red blood cell; WBC, white blood cell; N, neutrophil; L, lymphocyte; NLR, neutrophil-to-lymphocyte ratio; Hb, hemoglobin; PLT, platelet; PA, prealbumin; ALP, alkaline phosphatase; AFP, alpha fetoprotein.

**Table 3:** Univariate ordinal logistic analysis for MVI presence in the derivation cohort (N = 800).

Factor	M0 (N = 382)	M1 (N = 257)	M2 (N = 161)	Estimated	Sig.	95% Cl
	N or median (range)	N or median (range)	N or median (range)			
**Gender**						
Female (105)	49	30	26	0.137	0.484	(-0.246, 0.520)
Male (695)	333	227	135	0		
**Age (year)**						
≤40 (106)	37	39	30	0.737	0.001	(0.316,1.158)
40–60 (428)	205	131	92	0.261	0.080	(-0.031,0.552)
≥60 (266)	140	87	39	0		
**Hepatitis virus type**						
None (94)	54	25	15	-2.057	0.073	(-4.303, 0.189)
Hepatitis B (683)	313	229	141	-1.633	0.149	(-3.848, 0.583)
Hepatitis C (20)	14	3	3	-2.542	0.038	(-4.940, -0.144)
Hepatitis B+C (3)	1	0	2	0		
**Antiviral therapy**						
No (718)	347	228	143	-0.197	0.365	(-0.622, 0.229)
Yes (82)	35	29	18	0		
**HBsAg (+)**						
No (147)	79	40	28	-0.373	0.037	(-0.723, -0.023)
Yes (653)	303	217	133	0		
HBV-DNA	746.5 (0–4.5 × 10^7^)	846.5 (0–1.9 × 10^7^)	617.0 (0–1.4 × 10^9^)	2.655E-8	0.165	(-1.093E-8, 6.402E-8)
**HBV-DNA < 100 IU/ml**						
No (536)	244	178	114	0.264	0.070	(-0.022, 0.551)
Yes (264)	138	79	47	0		
**HBsAb**						
-(733)	345	235	153	0.387	0.119	(-0.100, 0.874)
+(67)	37	22	8	0		
**Anti-HCV**						
-(776)	367	253	156	0.462	0.258	(-0.339, 1.263)
+(24)	15	4	5	0		
**HBeAg**						
-(749)	361	237	151	-0.187	0.488	(-0.716, 0.342)
+(51)	21	20	10	0		
**HBeAb**						
-(232)	121	55	56	-0.313	0.056	(-0.635, 0.008)
+(568)	261	202	105	0		
**HBcAb**						
-(109)	59	13	37	-0.477	0.083	(-1.016, 0.062)
+(691)	323	244	124	0		
**With PH**						
No (671)	309	222	140	0.657	0.330	(-0.666, 1.981)
Mild (85)	45	29	11	0.320	0.649	(-1.058, 1.699)
Moderate (35)	22	5	8	0.158	0.833	(-1.310, 1.627)
Severe (9)	6	1	2	0		
**With SR**						
No (717)	351	221	145	-0.288	0.180	(-0.710, 0.133)
Yes (83)	31	36	16	0		
**CTP grade**						
A (764)	365	249	150	-0.363	0.846	(-4.029, 3.304)
B (36)	17	8	11	0		
**Tumor MD (cm)**	4.5 (1.0–21.0)	6.5 (1.2–33.0)	8.0 (2.0–20.0)	0.141	0.000	(0.108, 0.174)
**Tumor number**						
1 (558)	311	177	70	-1.907	0.000	(-2.295, -1.520)
2 (95)	40	35	20	-1.345	0.000	(-1.854, -0.836)
3 (24)	10	6	8	-1.061	0.011	(-1.875, -0.247)
≥4 (123)	21	39	63	0		
**Tumor location**						
left (187)	83	60	44	-0.490	0.062	(-1.005, 0.026)
right (546)	279	170	97	-0.788	0.001	(-1.258, -0.318)
bilobar (67)	20	27	20	0		
**With PVTT**						
No (723)	374	240	109	-3.158	0.000	(-4.667, -1.650)
Vp1 (3)	1	0	2	-1.181	0.385	(-3.847, 1.485)
Vp2 (22)	3	5	14	-0.939	0.284	(-2.657, 0.778)
Vp3 (41)	3	11	27	-0.759	0.360	(-2.386, 0.868)
Vp4 (11)	1	1	9	0		
**With BDTT**						
None (765)	376	248	141	-1.431	0.000	(-2.201, -0.661)
Microscopic (11)	1	2	8	1.012	0.189	(-0.497, 2.522)
Macroscopic (24)	5	7	12	0		
**With HVTT**						
None (776)	380	250	146	-1.754	0.073	(-3.674, 0.165)
Vv1 (3)	0	2	1	-.417	0.775	(-3.274, 2.440)
Vv2 (17)	2	3	12	0.542	0.624	(-1.626, 2.710)
Vv3 (4)	0	2	2	0		
**With IVCTT**						
No (794)	382	254	158	-1.692	0.035	(-3.262, -0.122)
Yes (6)	0	3	3	0		
**Tumor capsular**						
No (492)	213	167	112	0.477	0.001	(0.202, 0.753)
Incomplete (12)	5	5	2	0.405	0.462	(-0.674, 1.484)
Complete (296)	164	85	47	0		
**Lymph node metastasis**						
No (793)	381	254	158	-1.273	0.074	(-2.670, 0.124)
Yes (7)	1	3	3	0		
**ALT (U/L)**	36.6 (8.6–369.3)	39.0 (1.0–423.8)	38.7 (8.4–172.3)	0.001	0.545	(-0.002, 0.005)
**AST (U/L)**	37.0 (14.4–382.3)	43.0 (15.9–610.6)	48.6(19.8–349.3)	0.005	0.010	(0.001, 0.008)
**LDH ≥ 176.58 (U/L)**						
No (528)	300	168	60	-0.862	0.000	(-1.345, -0.379)
Yes (272)	82	89	101	0		
**ALP (U/L)**	102.0(46.0–538.8)	108.0 (40.2–493.0)	109.0(33.0–618.7)	0.001	0.451	(-0.002, 0.003)
**PA (mg/L)**	177.9(22.7–473.0)	182.0 (49.0–370.9)	168.0(30.0–394.0)	-0.001	0.370	(-0.003, 0.001)
**γ-GGT (U/L)**	70.2 (10.3–911.3)	78.1(12.4–1208.7)	88.2(12.9–1411.3)	0.001	0.040	(0.000, 0.002)
**5'-NT (U/L)**	10.6 (0.4–86.5)	10.4 (0.8–105.2)	11.9 (2.3–144.0)	0.010	0.046	(0.000, 0.020)
**TBA (μmol/L)**	6.6 (0–154.4)	4.7 (0–246.8)	6.1 (0.3–172.7)	0.003	0.383	(-0.004, 0.010)
**TP (g/L)**	64.2(45.5–87.6)	65.1 (39.3–81.9)	65.8 (48.0–81.0)	0.026	0.026	(0.003, 0.050)
**ALB (g/L)**	38.8(25.6–64.6)	39.9 (29.0–50.1)	39.6 (27.0–49.3)	0.038	0.024	(0.005, 0.072)
**GLB (g/L)**	25.4 (14.2–50.6)	25.8 (14.2–41.3)	16.1 (14.2–39.0)	0.005	0.474	(-0.009, 0.020)
**AG ratio**	1.5 (0.7–3.1)	1.5 (0.8–3.0)	1.5 (0.9–2.6)	0.035	0.873	(-0.398, 0.469)
**TBIL (mmol/L)**	15.0 (5.4–57.4)	16.1 (5.3–210.1)	15.9 (5.9–288.0)	0.008	0.462	(-0.962, 0.437)
**DBIL (mmol/L)**	5.1 (1.0–37.8)	5.1 (1.3–115.2)	5.2 (1.9–201.0)	0.012	0.101	(-0.002, 0.027)
**RBC (×10^12^)**	4.3 (1.9–7.5)	4.4 (2.5–6.8)	4.5 (2.5–6.5)	0.386	0.001	(0.163, 0.610)
**WBC (×10^9^)**	5.4 (1.3–22.1)	5.4 (2.0–16.1)	5.8 (2.1–20.3)	-0.002	0.960	(-0.064, 0.061)
**N**	3.3 (0.8–19.6)	3.3 (0.9–14.3)	3.7 (1.0–17.9)	0.023	0.527	(-0.047, 0.093)
**L**	1.4 (0.3–3.2)	1.3 (0.4–3.7)	1.2 (0.4–3.6)	-0.309	0.048	(-0.616, -0.002)
**NLR**	2.3 (0.5–19.4)	2.6 (0.8–16.0)	2.9 (1.0–10.0)	0.047	0.217	(-0.028, 0.123)
**Mono**	0.5 (0.1–1.6)	0.5 (0.1–1.1)	0.5 (0.2–1.2)	-0.101	0.798	(-0.870, 0.669)
**MLR**	0.3 (0.04–2.0)	0.3 (0.03–1.0)	0.4 (0.1–1.4)	0.284	0.475	(-0.494, 1.061)
**Hb (g/L)**	135.0(67.0–196.0)	138.0 (80–199)	137.0 (84–190)	0.009	0.023	(0.001, 0.016)
**Hct**	41.1 (20.9–59.6)	41.5 (24.3–61.6)	41.7 (25.1–58.9)	0.022	0.110	(-0.005, 0.050)
**PLT > 162.5 × 10^12^**						
Yes (278)	114	92	72	-0.588	0.000	(-0.843, -0.332)
No (522)	268	165	89	0		
**PDW**	15.0 (8.8–72.5)	14.1 (0.3–72.4)	13.6 (0.9–83.5)	-0.005	0.441	(-0.017, 0.007)
**PCT**	0.2 (0.04–0.5)	0.2 (0.04–1.1)	0.2 (0.04–0.5)	0.127	0.467	(-0.214, 0.467)
**PT > 17s**						
No (755)	359	242	154	0.410	0.608	(-1.159, 1.979)
Yes (45)	23	15	7	0		
**APPT > 40s**						
No (715)	340	227	148	0.231	0.297	(-0.203, 0.664)
Yes (85)	42	30	13	0		
**TT > 21s**						
No (699)	328	224	147	0.176	0.435	(-0.266, 0.618)
Yes (101)	54	33	14	0		
**INR > 1.5**						
No (790)	377	255	158	-0.620	0.634	(-3.170, 1.931)
Yes (10)	5	2	3	0		
**FDP**	1.7 (0.1–100.0)	1.8 (0.0–68.1)	2.2 (0.1–91.2)	0.021	0.071	(-0.002, 0.044)
**FIB (g/L)**	2.3 (1.2–7.7)	2.6 (0.9–6.4)	3.2 (1.2–7.7)	0.322	0.000	(0.174, 0.470)
**DD**	0.4 (0.02–31.9)	0.4 (0.01–9.7)	0.7 (0.08–18.9)	0.084	0.062	(-0.004, 0.173)
**BUN (mmol/L)**	4.7 (2.4–11.1)	4.6 (1.9–11.2)	4.3 (2.1–13.6)	-0.087	0.124	(-0.199, 0.024)
**Cr (mmol/L)**	66.1 (32.3–170.6)	67.0 (31.8–171.5)	63.2(36.9–207.0)	-0.001	0.806	(-0.010, 0.008)
**AFP ≥ 11.08 (μg/L)**						
No (269)	186	55	28	-0.887	0.000	(-1.170,-0.603)
Yes (531)	196	202	133	0		
**Edmondson-Steiner stage**						
I (24)	20	1	3	-1.758	0.002	(-2.874, -0.643)
II (255)	142	56	57	-0.355	0.145	(-0.832, 0.123)
III (444)	188	170	86	-0.022	0.922	(-0.473, 0.428)
IV (77)	32	30	15	0		
**Metavir inflammation grade**						
A0 (65)	33	18	14	0.523	0.308	(-0.484, 1.530)
A1 (523)	244	183	96	0.648	0.161	(-0.258, 1.554)
A2(192)	92	52	48	0.738	0.120	(-0.191, 1.668)
A3 (20)	13	4	3	0		
**Metavir fibrosis grade**						
F0 (18)	10	5	3	-0.006	0.989	(-0.937, 0.924)
F1 (54)	27	16	11	0.229	0.430	(-0.340, 0.797)
F2 (315)	138	110	67	0.419	0.014	(0.083, 0.754)
F3 (208)	92	73	43	0.396	0.034	(0.030, 0.763)
F4 (205)	115	53	37	0		

HR, hazard ratio; Dis-course, disease course; DM, diabetes mellitus; HV, hepatitis virus; MD, maximum diameter; PH, portal hypertension; SR, spontaneous rupture; BS, blood sugar; PVTT, portal vein tumor thrombosis; BDTT, bile duct tumor thrombosis; HVTT, hepatic vein tumor thrombosis; IVCTT, inferior vena cava tumor thrombosis; ALT, glutamic pyruvic transaminase; AST, aspartate aminotransferase; ALP, alkaline phosphatase; PA, prealbumin; γ-GGT, γ-glutamyl transpeptidase; 5’-NT, nucleotidase; LDH, lactate dehydrogenase; TBA, total bile acid; TP, total protein; ALB, albumin; GLB, globulin; AG, albumin-to-globulin ratio; TBIL, total bilirubin; DBIL, direct bilirubin; RBC, red blood cell; WBC, white blood cell; N, neutrophil; L, lymphocyte; NLR, neutrophil-to-lymphocyte ratio; MONO, monocyte; MLR, monocyte-to-lymphocyte ratio; Hb, hemoglobin; PLT, platelet; PLR, platelet-to-lymphocyte ratio; PT, prothrombin time; APTT, activated partial thromboplastin time; TT, thrombin time; FIB, plasma fibrinogen; D-D, D-dimer; AFP, alpha fetoprotein. OR, odds ratio = e ^Estimate^.

**Table 4:** Multivariable analysis of predictors associated with MVI.

Factor	Estimate	SE	Wald	df	Sig.	OR	95% CI	
Tumor MD (cm)	0.101	0.042	5.848	1	0.016	1.106	(1.106, 1.019)	
Tumor number								
1	-3.992	1.576	6.413	1	0.011	0.018	(-7.082, -0.902)	
2	-2.865	1.660	2.981	1	0.084	0.057	(-6.118, 0.387)	
3	-2.675	1.930	1.922	1	0.166	0.069	(-6.457, 1.107)	
≥4	0			0		1		
LDH ≥ 176.58 (U/L)								
No	-0.749	0.344	4.736	1	0.030	0.473	(-1.423, -0.074)	
Yes	0			0		1		
γ-GGT (U/L)	0.004	0.002	5.699	1	0.017	1.004	(1.004, 1.001)	

OR (odds ratio) = e^Estimate^; CI, confidence interval; MD, maximum diameter; LDH, lactate dehydrogenase; γ-GGT, γ-glutamyl transpeptidase.

### Establishment and Validation of an M2 Predictive Nomogram

**Figure 2:**
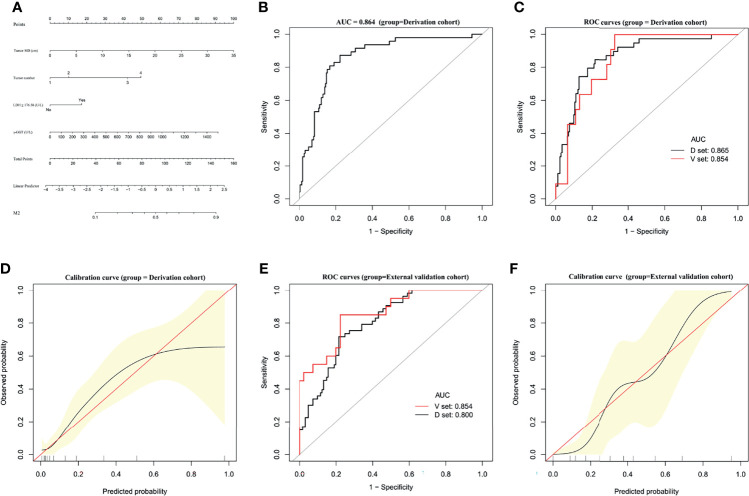
Establishment and validation of M2 predictive nomogram for HCC patients. **(A)** M2 prediction scores are calculated with this four-factor nomogram by finding the position and corresponding y-axis point value of each variable and summing the point values of all five variables, and then reading the probability on the x-axis. **(B)** M2 nomogram AUC = 0.864 (95% CI, 0.8081–0.9196). **(C)** Bootstrap analysis for internal validation and **(D)** GiViTI calibration plots showing good prediction-observation agreement for M2 in the derivation cohort. **(E)** Bootstrap analysis for internal validation and **(F)** GiViTI calibration plots showing good prediction-observation agreement for M2 in the external validation cohort. AUCs are shown in the figure and reported with 95% CIs in the text together with the c-index. Calibration plots (black lines) show fitted polynomial logistic function curves of the relationship between the logit transformation of the predicted probabilities and empirical outcomes (shaded yellow, 95% CI). Ideal reference lines are red. HL chi-square calibration values for each cohort are reported in the Results text.

A predictive nomogram for M2 developed based on our multivariate analysis results is presented in **Figure 2A**. M2 prediction scores calculated from this nomogram consisted of the total points for each patient obtained by adding the points for each of the five analyzed factors. The AUC of the nomogram to predict M2 were 0.864 (95% CI, 0.8081–0.9196) **(** **Figure 2B** **)**. In bootstrap analysis for internal validation of the derivation cohort data, we obtained AUCs for M2 prediction of 0.865 (95% CI, 0.8038–0.9234) for the development dataset (D-set) and 0.854 (95% CI, 0.7541–0.9534) for the validation dataset (V-set) **(** **Figure 2C** **)**, with a c-index of 0.817 (95% CI, 0.774–0.860) indicating that the nomogram had good performance for distinguishing between M2 and non-M2 patients. A GiViTI calibration plot showed good consistency between the observed frequency and predicted probability for M2 among patients in the derivation cohort **(** **Figure 2D** **)**. The HL chi-square calibration value of the M2 model was 7.601 for the derivation cohort (*p* = 0.473).

In the bootstrap analysis for internal validation performed with the external validation cohort data, AUCs for M2 prediction were 0.800 (95% CI, 0.7190–0.8517) for the D-set and 0.854 (95% CI, 0.7933–0.9221) for the V-set **(** **Figure 2E** **)**, with a c-index of 0.772 (95% CI, 0.715–0.828). A GiViTI calibration plot affirmed that the model calibration yielded good consistency between the observed frequency and predicted probability for among patients with M2-class MVI in the external validation cohort **(** **Figure 2F** **)**, with an HL chi-squared calibration M2 prediction value of 10.659 for the external validation cohort (*p* = 0.222).

### Establishment and Validation of an M1 Predictive Nomogram

**Figure 3:**
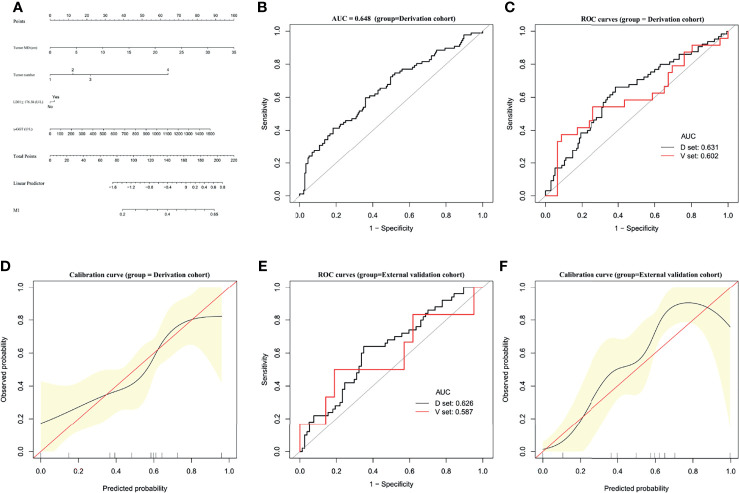
Establishment and validation of M1 predictive nomogram for HCC patients**. (A)** M1 prediction scores were calculated with this four-factor nomogram by finding the position and corresponding y-axis point value of each variable and summing the point values of all five variables, and then reading the probability on the x-axis. **(B)** M1 nomogram, AUC = 0.648 (95% CI 0.5773–0.7197). **(C)** Bootstrap analysis for internal validation and **(D)** GiViTI calibration plots showing good prediction-observation agreement for M1 in the derivation cohort. **(E)** Bootstrap analysis for internal validation and **(F)** GiViTI calibration plots showing good prediction-observation agreement for M1 in the external validation cohort. AUCs (shown in the figure and reported with 95% CI and c-index values in the text) indicate fair discrimination but with insufficient separation, yielding poor discrimination. HL chi-square calibration values for each cohort are reported in the Results text.

A predictive nomogram was constructed for predicting M1 status based on the results of our multivariate analysis **(** **Figure 3A** **).** The AUC of the predictive model for M1 diagnosis was 0.648 (95% CI, 0.5773–0.7197) (**Figure 3B**). In bootstrap analysis for internal validation, the AUCs for M1 prediction in the D-set and V-set were 0.631 (95% CI, 0.5473–0.7149) and 0.602 (95% CI, 0.5412–0.7533), respectively (**Figure 3C**). A GiViTI calibration plot showed good consistency between the observed frequency and predicted probability for M1 among patients in the derivation cohort **(** **Figure 3D** **)**, the c-index was 0.623 (95% CI, 0.551–0.696). The HL chi-squared calibration values for M1 prediction were 5.284 (derivation cohort, *p* = 0.727).

In the bootstrap analysis for internal validation performed with the external validation cohort data, AUCs for M1 prediction were 0.626 (95% CI, 0.5026–0.7109) for the D-set and 0.587 (95% CI, 0.4827–0.6854) for the V-set **(** **Figure 3E** **)**, with a c-index of 0.623 (95% CI, 0.551–0.696). A GiViTI calibration plot affirmed that the model calibration yielded good consistency between the observed frequency and predicted probability for among patients with M1-class MVI in the external validation cohort **(** **Figure 3F** **)**, with an HL chi-squared calibration M1 prediction value of 4.523 for the external validation cohort (*p* =0.807).

These results indicate that there was fair discrimination, and the risk of disease was relatively close across groups and not readily distinguishable among M1-class and non-M1 MVI class, although the model has satisfactory calibration. These data do not support the effectiveness of the M1 predictive nomogram in clinical applications.

### Establishment and Validation of an M0 Predictive Nomogram

**Figure 4:**
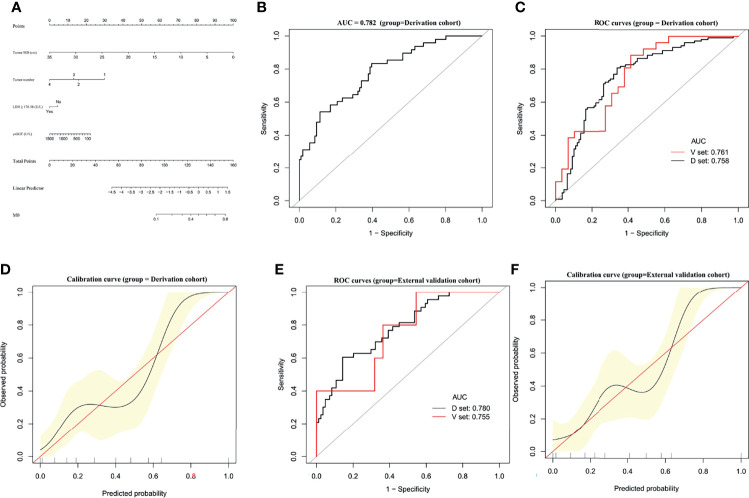
Establishment and validation of M0 predictive nomogram for HCC patients. **(A)** M0 prediction scores are calculated with this four-factor nomogram exactly as in **Figure 2A**. **(B)** M0 nomogram AUC = 0.782 (95% CI, 0.7044–0.8597). **(C)** Bootstrap analysis for internal validation and **(D)** GiViTI calibration plots showing good prediction-observation agreement for M0 in the derivation cohort. **(E)** Bootstrap analysis for internal validation and **(F)** GiViTI calibration plots showing good prediction-observation agreement for M0 in the external validation cohort. AUCs are shown in the figure and reported with 95% CIs in the text together with the c-index. GiViTI calibration plots showing good prediction-observation agreement for M0 in the derivation **(D)** and external validation **(F)** cohorts. Calibration plots (black lines) show fitted polynomial logistic function curves of the relationship between the logit transformation of the predicted probabilities and empirical outcomes (shaded yellow, 95% CI). Ideal reference lines are red. HL chi-square calibration values for each cohort are reported in the Results text.

A predictive nomogram was constructed for predicting M0 status based on the results of our multivariate analysis (**Figure 4A**). The AUC of the M0 predictive nomogram was 0.782 (95% CI, 0.7044–0.8597) (**Figure 4B**). In bootstrap analysis for internal validation, the AUCs for M0 prediction in the D-set and V-set were 0.758 (95% CI, 0.6921–0.8269) and 0.761 (95% CI, 0.6348–0.8877) (**Figure 4C**), respectively, the c-index was 0.751(95% CI, 0.706–0.797). A GiViTI calibration belt plot was generated and demonstrated model calibration with good consistency between the observed frequency and predicted probability of patients with an M0 status in the derivation cohort **(** **Figure 4D** **)**. The HL chi-squared calibration value for M0 prediction was 9.213 for the derivation cohort (*p* = 0.325). In bootstrap analysis for internal validation of the external validation cohort, the AUCs for M0 prediction in the D-set and V-set were 0.780 (95% CI, 0.6978–0.8428) and 0.755 (95%CI, 0.6688-0.8574) **(** **Figure 4E** **)**, respectively, with a c-index was 0.732 (95% CI, 0.685–0.779). A GiViTI calibration plot was generated and demonstrated that the model calibration showed good consistency between the observed frequency and predicted probability of M0 status among patients in the external validation cohort **(** **Figure 4F** **)**. The HL chi-square calibration value of the M0 model was 5.690 for the external validation cohort (*p* = 0.682). The M0 predictive nomogram showed good performance in distinguishing patients with versus not with M0 status.

## Discussion

The present multicenter study provides a comprehensive review of clinical data from hepatectomized HCC patients treated at two Chinese hepatobiliary centers. This work addresses the necessity to differentiate MVI classification before making treatment strategy in this patient population.

Extensive studies have been conducted to explore useful clinical/tumor/radiological factors for prediction of MVI status. Most have been single-center cohort studies with relatively small sample sizes, characteristics which may impede their reliability and limit the generalizability of their conclusions (5). It is noteworthy that Lei et al. published a large cohort study (N = 1004) of patients with HCC (diagnosed in accordance with the Milan criteria) (28); their cohort consisted mainly of patients with HBV-related HCC and had characteristics similar to ours. Employing an MVI presence/absence classification, the authors constructed a nomogram with unadjusted C index values similar to those obtained for our M2 prediction models: 0.81 (95%CI, 0.78–0.85) in their derivation cohort versus 0.817 (95% CI, 0.774–0.860) in ours; and 0.80 (95%CI, 0.75–0.86) in their validation cohort versus 0.772 (95% CI, 0.715–0.828) in ours. Methodological differences between Lei et al.’s study and ours precludes direct comparison. It appears likely to us that the MVI presence group in Lei et al.’s study included M1 and M2 diagnoses. It is difficult to distinguish between M1 and M2 based on that study’s conclusions and thus to make deductions about the true role of MVI in clinical HCC management strategy and prognosis evaluation.

In another recent relatively large two-center retrospective cohort study conducted in China, Zhang et al. (29) adopted MVI classification criteria that were the same as ours. In their study, they predicted MVI status based on a combination of computed tomography radiomics data and two clinical factors: age and alpha-fetoprotein test result (positive/negative). They obtained AUCs of 0.806, 0.803, and 0.796 in their training, test, and independent validation cohorts, respectively. Although they used MVI classification criteria delineating M0, M1, and M2, they did not provide data on prediction of M1 or M0. Based on AUCs for prediction, our results for M2 are likely comparable with Zhang et al.’s MVI risk prediction for MVI positivity/negativity; C-index values were not reported in Zhang’s study. Importantly, simple MVI positivity cannot be regarded as being indicative of M2 status, and there were no robust data differentiating M0 or M1 that could be inferred from Zhang’s model.

MVI classification has not received sufficient attention in prognosis evaluation for patients with HCC. In a recent multicenter study, researchers from the Liver Cancer Pathology Group of China (30) used MVI classification data to analyze postoperative prognosis in 2,573 HCC patients who underwent curative hepatectomy. They found that the 3-year postoperative recurrence rates in their M0, M1, and M2 groups were 62.5%, 71.6%, and 86.1%, respectively (p < 0.001), with corresponding 3-year overall survival rates of 94.1%, 87.5% and 67.0%, respectively (p < 0.001). M1 grade was associated with early recurrence, whereas M2 grade was associated with both early and late recurrence. Our current study expands upon the prior data with a focus on MVI classification and further addresses the importance of MVI classification in evaluating the potential importance of MVI in HCC patient prognosis.

The main strength of the present study is that we developed models for predicting M0, M1, and M2 disease classifications. We found that only the M0 and M2 models could be validated as clinically applicable for use in patients undergoing hepatectomy for HCC. Our nomogram models demonstrated good prediction abilities for M0 and M2 and yielded good consistency between predicted probabilities of MVI and empirical frequency of MVIs in both internal and external validation cohorts. According to the MVI classification criteria used in our study, the clinicopathological factors selected by multivariate analysis showed unsatisfactory discrimination for the M1 prediction model, despite the model showing good calibration. Also, very few clinicopathological variables harvested meaningful cutoff point values determined by ROC analysis for M1 diagnosis.

Given the unsatisfactory performance of the M1 nomogram in the present study, and our previous findings showing no difference in early recurrence after curative hepatectomy between M0 and M1 class HCC patients (10), it may be that MVI only has measurable effects on prognosis (postoperative recurrence, overall survival) when it reaches M2 class severity. The present study addresses the question of whether the M1 class distinction provides any advantage. When MVI is classified simply as absent (M0) or present (M1/M2), patients with M1 disease might receive overtreatment, such as unnecessary postoperative adjunct therapies, and inaccurate prognostic information given that clinical demonstrations have shown that adjuvant transarterial chemoembolization can improve overall and disease-free survival in HCC patients with MVI (31). The significance of MVI class is also relevant for HCC management with respect to deciding whether a particular patient should have local ablation, non-anatomical surgical resection, or anatomical surgical resection, and appropriate margin widths. A notable strength of our study is that we included complex and diverse HCC cases, augmenting the reliability and applicability of the results. It seems reasonable to presume that the incidence of MVI, especially M2, should be positively related to an advanced state of HCC with more invasive features. However, our current findings do not support such a view. We speculate that HCC biological characteristics related to MVI appear to differ from those related to macrovascular invasion and thus may have a distinct influence on prognosis. The molecular biological mechanisms of such a distinct influence remain to be clarified. Interestingly, given the previous studies which would suggest otherwise (16, 32), we were surprised that preoperative HBV-DNA level was not found to be significantly associated with MVI in our HCC patients.

To date, various MVI classification schema have been proposed (11–17) and no consensus on an MVI classification scheme has been reached. We favor the strategy of establishing an MVI classification schema that takes into account the severity of MVI burden, which may include the number of malignant cells in an embolus, the presence or absence of vessel wall invasion, and distance from the primary lesion. Tumor size and number have been identified as risk factors in previous reports, albeit with different cut-off values (6). The presently obtained ROC curve analysis-based tumor size cut-off values for M1 (5.55cm) and M2 (6.95 cm) were quite close. A previous meta-analysis found that MVI was detected in about 25% of small liver cancer cases (<2 cm) (33). In the present study, among cases with a solitary HCC with a diameter ≤ 2 cm, M1 and M2 classification rates for the derivation cohort were 15.8% (9/57) and 3.5% (2/57), while M1 and M2 classification rates for the external validation group were 9.1% (1/11) and 0 (0/11), respectively. In our multivariate analysis, tumor size was inputted as a continuous variable to minimize information loss, thereby reflecting the value of tumor size for MVI prediction. With respect to tumor number, we were only able to harvest a clinically useful cut-off value from our ROC analysis for M2 prediction (≥1.5, effectively ≥2).

In this study, we also considered the number of satellite neoplastic lesions, which may be more reliable for preoperative imaging evaluation. HCC satellite lesions are detached micrometastatic nodules of HCC cells that have become embedded within the hepatic parenchyma, differing from MVI lesions in that they are thought to represent direct tissue invasion by malignant cells. Hence, MVI prediction models based on radiology examination alone may be intrinsically limited.

Several findings that have not been reported in previous studies are presented in this research report. Historically, serum LDH has been an indicator of cell injury and necrosis, and thus may reflect tumor burden. In a meta-analysis, preoperative LDH was shown to be significantly associated with poor prognosis in patients with HCC (34). However, further analysis showed that serum LDH levels correlated inversely with checkpoint inhibitor treatment responsivity, suggesting that the prognostic attributes of elevated LDH levels may go beyond tumor burden alone (35). High LDH levels could be consequent to elevated glycolytic activity of tumor cells and hypoxia-associated tumor necrosis; both glycolysis and hypoxia contribute to the immuno-suppressiveness of microenvironments (36). Elevation of the glycolysis metabolite lactate due to increased serum LDH can influence the complex metabolic crosstalk between tumor cells and tumor-infiltrating immune cells in the tumor microenvironment in multiple ways (37, 38). Additionally, lactate increases acidify the tumor microenvironment, thereby promoting multiple critical oncogenic processes, including angiogenesis, tumor cell invasion, and metastasis, while suppressing anticancer immunity (33, 39). Regarding the present finding showing that an LDH serum level ≥ 176.58 U/L was independently associated with MVI, it is interesting to note that this cut-off value is within the normal range for LDH (100.0–240.0 U/L). Moreover, we did not assess different LDH isoforms separately because serum LDH isoform analysis is not regularly performed in this patient population. In addition, the relationship between serum LDH levels and tumor LDH expression remains unclear, and the mechanism by which either may influence MVI are not known. Notwithstanding, our findings indicate that LDH levels may play an important role in MVI prediction in patients with HCC.

γ-GGT alone or combination with other markers has been extensively explored as a prognostic factor in HCC patients (40–44). Here, we found that serum γ-GGT level was a significant predictor of MVI, a finding that has not been reported previously. High serum levels of γ-GGT prior to treatment have been reported to correlate with poor survival as well as with unfavorable clinicopathological characteristics in patients diagnosed with HCC (45). For a detailed discussion of the potential mechanisms by which serum γ-GGT level may have prognostic value in patients with HCC, the reader is referred to Sun and colleagues’ meta-analysis of 18 studies from China and 2 studies from western countries (45). Since Sun’s review was published, subsequent γ-GGT studies of HCC have been conducted primarily in HBV-endemic areas (40, 41, 43, 44). It would be of interest to determine whether γ-GGT may be a more valuable marker in HBV-related HCC than in non-HBV-related HCC. We speculate that γ-GGT level might reflect the formation of a microenvironment within which oxidative stress promotes tumor cells to produce γ-GGT, contributing, to some extent, to MVI formation. However, the exact direct mechanisms of elevated γ-GGT in cancer initiation and progression have not been resolved. The question of whether such effects of γ-GGT level on MVI occur with HCC arising from other etiologies deserves further investigation.

Prior studies that have examined preoperative radiological evaluation of MVI status have been criticized fairly for having relatively small sample sizes, using varying imaging modalities and radiological parameters, and using advanced imaging technologies with limited utility in clinical practice. These drawbacks have precluded consensus and the establishment of valuable information to guide preoperative accurate MVI prediction in clinical practice. Despite being far from consensus, imaging evaluation approaches remain promising for MVI prediction and worthy of further research (46). In a recent meta-analysis of pooled data (46), diagnostic AUC values for radiomics and non-radiomics factors were similar (0.8550 and 0.8601, respectively). Our proposed model for M2 prediction outperformed these radiomics or non-radiomics methods slightly in terms of the simple index of AUC (0.864).

A major strength of our study is the inclusion of prediction models for M0 and M1. This valuable information cannot be acquired from the studies included in Huang’s meta-analysis (46) because included studies dichotomized MVI status as present or absent. Overlooking these factors could affect the judgment of MVI status based on preoperative imaging tools. We have noticed several shortcomings when radiological and/or radiomics findings alone are used for MVI prediction model construction. Firstly, tumor characteristics inferred from preoperative imaging, including tumor size and number, may deviate from pathological findings. Secondly, the accuracy and practicality of radiomics models are questionable due to the lack of standardization in radiomics. Depending on the subjective judgment of particular diagnostic radiologists, some parameters may be overly specialized.

With respect to the clinical relevance of the present findings, it is important to note that our models involve variables that are easily detectable. Moreover, the fact that a large dataset was gathered from more than one center is an added strength in that it makes our conclusions more generalizable. The medical data that we analyzed were collected under real-world clinical conditions, providing high external validity and high generalizability. Our nomograms allowed reliable prediction of M0 and M2 for patients undergoing hepatectomy, and thus can be used to support preoperative treatment planning for patients with HCC.

## Study Limitations

Firstly, due to the retrospective nature of the study, potential selection bias might exist, causing an unbalanced number of patients in different MVI classifications. To overcome this limitation to some extent, cases were selected consecutively to reduce bias. Secondly, this study was conducted in a region where HBV is endemic. Therefore, the applicability of the results may be limited to a certain extent. Despite these limitations, this study provides notable insights that can help surgeons and clinicians determine appropriate treatment strategies for patients with HCC.

## Conclusion

We have developed and validated an MVI prediction model for patients with HCC. The model demonstrates excellent discriminative ability for M0 and M2 classes of MVI diagnosis. The present findings provide guidance information that clinicians can use in practice to improve preoperative treatment strategy decisions.

## Data Availability Statement

The original contributions presented in the study are included in the article/supplementary material. Further inquiries can be directed to the corresponding authors.

## Ethics Statement

The studies involving human participants were reviewed and approved by Hunan Provincial People’s Hospital (HPPH) and Affiliated Hospital of North Sichuan Medical College (NSMC). (HPPH approval no: 2020-018; NSMC approval no: 2020-055). The ethics committee waived the requirement of written informed consent for participation.

## Author Contributions

Study concept and design: WX and JL. Acquisition of data: YW, ZY, RL, and FL. Analysis and interpretation of data: WX, YW, ZY, and JL. Drafting of the manuscript: WX, YW, ZY, and JL. Statistical analysis: ZY, RL, and FL. Study supervision: WX and JL. All authors contributed to the article and approved the submitted version.

## Funding

This study was supported by funds from the Health Commission (project no. 20200074) and Education Department (project no. 20A313) of Hunan Province.

## Conflict of Interest

The authors declare that the research was conducted in the absence of any commercial or financial relationships that could be construed as a potential conflict of interest.

## Publisher’s Note

All claims expressed in this article are solely those of the authors and do not necessarily represent those of their affiliated organizations, or those of the publisher, the editors and the reviewers. Any product that may be evaluated in this article, or claim that may be made by its manufacturer, is not guaranteed or endorsed by the publisher.
